# The clinical phenotype of Israeli patients with Q703K mutation in the NLRP3 gene

**DOI:** 10.1186/1546-0096-13-S1-P42

**Published:** 2015-09-28

**Authors:** M Lidar, A Livneh, I Ben Zvi, R Cohen, Y Berkun, P Hashkes, H Peleg, A Kessel, R Almog, L Kali, G Slobodin, M Rozenbaum, Y Shinar

**Affiliations:** 1 Sheba medical center, Rheumatology unit, Ramat gan, Israel; 2 Sheba Medical Center, Tel Hashomer, Israel; 3 Hadassah Medical Center, Jerusalem, Israel; 4 Shaare Zedek Medical Center, Jerusalem, Israel; 5 Bnai Zion Medical Center, Haifa, Israel

## Background

Cryopyrin associated periodic syndromes (CAPS) comprise a spectrum of autoinflammatory disorders of varying severity caused by mutations in the NLRP3 gene. The Q703K allele, reaching 5% of the total allele count in the general population, is considered either functional polymorphism or a low penetrance mutation

## Aim

To describe the clinical phenotype of the Israeli patients in whom the Q703K allele was found.

## Methods

Ten patients carrying the Q703K mutation were identified among 70 patients in whom the diagnosis of CAPS was suspected on clinical grounds.

## Results

Seven female and 3 male patients with a mean age of 22.5±17.8 years and a mean diagnosis delay of 12.4 years were identified. Their clinical characteristics ranged from self resolving attacks of fever, urticaria and arthralgia to a chronic, debilitating steroid-dependent inflammatory disease. Splenomegaly, transfusion-dependent anemia, sensory neuropathy and pericarditis, manifestations which are not included in the traditional CAPS-spectrum, were detected in some of the patients. The majority of patients responded to high dose steroid therapy. DMARDS such as methotrexate, azathioprine and colchicine were generally ineffective at reducing steroid dose or attack rate. Therapy with TNF inhibitors or anti IL-1 agents was instituted in 4 patients with a favorable response. All but one patient needed chronic anti-inflammatory therapy to prevent attacks and reduce steroid dose.

## Conclusions

Our cohort of patients with the Q703K mutation, the largest reported to date, show a heterogeneous inflammatory phenotype, in which a CAPS component may be appreciated. Both IL-1 and TNF inhibitors seem to be effective in treating steroid resistant manifestations of this subgroup of CAPS patients.

